# Micromachining of Biolox Forte Ceramic Utilizing Combined Laser/Ultrasonic Processes

**DOI:** 10.3390/ma13163505

**Published:** 2020-08-08

**Authors:** Basem M. A. Abdo, Syed Hammad Mian, Abdualziz El-Tamimi, Hisham Alkhalefah, Khaja Moiduddin

**Affiliations:** 1 Advanced Manufacturing Institute, King Saud University, Riyadh 11421, Saudi Arabia; smien@ksu.edu.sa (S.H.M.); halkhalefah@ksu.edu.sa (H.A.); khussain1@ksu.edu.sa (K.M.); 2 Industrial Engineering Department, College of Engineering, King Saud University, P.O. Box 800, Riyadh 11421, Saudi Arabia; atamimi@ksu.edu

**Keywords:** biolox forte ceramic, laser beam machining, rotary ultrasonic machining, combined process, micromachining, microchannels, surface roughness, tool wear

## Abstract

Micromachining has gained considerable interest across a wide range of applications. It ensures the production of microfeatures such as microchannels, micropockets, etc. Typically, the manufacturing of microchannels in bioceramics is a demanding task. The ubiquitous technologies, laser beam machining (LBM) and rotary ultrasonic machining (RUM), have tremendous potential. However, again, these machining methods do have inherent problems. LBM has issues concerning thermal damage, high surface roughness, and vulnerable dimensional accuracy. Likewise, RUM is associated with high machining costs and low material-removal rates. To overcome their limits, a synthesis of LBM and RUM processes known as laser rotary ultrasonic machining (LRUM) has been conceived. The bioceramic known as biolox forte was utilized in this investigation. The approach encompasses the exploratory study of the effects of fundamental input process parameters of LBM and RUM on the surface quality, machining time, and dimensional accuracy of the manufactured microchannels. The performance of LRUM was analyzed and the mechanism of LRUM tool wear was also investigated. The results revealed that the surface roughness, depth error, and width error is decreased by 88%, 70%, and 80% respectively in the LRUM process. Moreover, the machining time of LRUM is reduced by 85%.

## 1. Introduction

Micromachining has acquired tremendous interest, as microcomponents can be seen in a broad array of applications, notably in the automotive, aerospace, electronics, green energy, and biomedical sectors [1]. Such microproducts or systems are typically made from difficult-to-machine materials, such as ceramics, metals, polymers, composites, etc., and represent intricate shapes [2]. The microcomponents consist of microfeatures such as microchannels, microholes, micropockets, etc., the accuracy of which is crucial to their effectiveness [3]. For example, the dimensional accuracy of microchannels plays a pivotal role in biomedical applications such as microfluidic systems [4]. The manufacturing of the microchannels in bioceramic materials has always been a tedious task and it is often challenging to refine and form them efficiently and effectively using traditional processing methods. Bioceramic materials are considered hard to machine by conventional methods of turning, milling, drilling, etc. due to their inherent characteristics including high hardness and brittleness along with low fracture toughness resulting in excessive wear of the tool, high material cracking, low efficiency and high machining costs. Besides, properties like chemical inertness and low electrical conductivity make bioceramics machining a challenge through the use of electrical and chemical processes. As a consequence, there is a restriction in commercial applications for bioceramic materials due to the high machining costs.

Laser beam machining (LBM) has been one of the nontraditional technologies of machining that can be extended to almost all materials heedless of their mechanical, electrical, and thermal properties [5]. Compared to conventional machining approaches, it has many benefits, including high versatility in machining complex shapes, contactless material removal, no tool wear, simple and inexpensive micromachining, etc. In LBM, the material is extracted using a high-power laser pulsed at a particular spot on the object to be machined. Numerous studies have highlighted the feasibility of using LBM to produce microfeatures on an array of materials including metals [6,7], alloys [8,9,10], polymers [11,12,13,14], glass [15,16,17], and ceramics [18,19,20,21,22]. For example, Leitz et al. [23] illustrated the laser ablation process of the metals and concluded that the pulse duration of the laser source seriously impacts its output. They reported that the nanosecond laser system resulted in maximum ablation efficiency, while the ablation performance of the pico- and femtosecond system were considerably lower. As stated by Gaudiuso et al. [24], the pulse repetition, scan rate, pulse interval, and pulse intensity are the critical variables that would strongly affect the laser cutting quality and functionality. Thus, a viable solution to the processing parameters is essential to get efficient and effective machining through laser systems. To facilitate the femtosecond laser processing of the sintered alumina surface, Oosterbeek et al. [25] undertook the optimization of laser processing parameters. The focal length and depth, laser power, percentage of passes, and material translation velocity were optimized for the removal rate and better performance in this analysis. This operation greatly increased machining speeds of alumina surface, thereby minimizing the costs, and making femtosecond laser machining a feasible choice for industrial consumers. Olbrich et al. [26] also explored the ablation of thin metal films using variable-pulsed laser radiations. They studied the ablation characteristics of several metals, namely aluminum, gold, molybdenum, nickel, and platinum focusing on pulse duration for single-pulsed ultrafast laser radiation. Similarly to LBM, rotary ultrasonic machining (RUM) has also shown its capabilities for producing microfeatures in hard-to-cut materials such as advanced ceramics, glasses, and alloys. RUM can be described as a hybrid process combining the material removal of both diamond grinding and stationary ultrasonic machining (USM) [27]. It is a purely mechanical and precise method of machining, primarily used for finishing and microproduction. RUM is frequently involved in the production of hard and brittle materials like ceramics [28,29], silicon [30], composites [31], glass [32,33] and crystals [34]. In RUM, a higher material removal rate (MRR) can be reached as opposed to diamond grinding or USM [35]. RUM also has characteristics of superior surface finish, lower cutting forces, and tool wear, as well as hardly any constraint due to the electrical or chemical properties of substrate materials [36]. Besides, the mechanical and metallurgical properties of RUM are not disturbed, and there is no thermal damage to the work material [37].

Researchers are attracted to establish hybrid micromachining processes (HMMP) in response to challenges in the micromachining realm and to resolve the shortcomings of individual LBM and RUM processes. In HMMP, two or more processes for machining are coupled to maximize the benefits of constituent processes while reducing their detrimental implications when implemented independently [38]. For example, in the published paper [39], a hybrid of laser-assisted micromilling was applied to produce microscale grooving on H-13 hard steel. Findings demonstrated that the precision of the groove depth was boosted with the assistance of laser heating—the cutting force and the surface roughness decreased by 17% and 36% respectively. Similarly, the hybrid of laser and diamond grinding was introduced by Fortunato et al. [40] to grind silicon nitride. Analysis indicated a drop of 30 percent in cutting force while using the hybrid method compared to the cutting force of single-diamond grinding. In another study [41], a laser water-jet hybrid process was employed to machine silicon carbide materials. Al-Ahmari et al. [42] utilized the electric-discharge machining (EDM) process to finalize the microholes that were premachined by laser processing in nickel–titanium material. Laser-guided grinding of zirconia ceramic material was also included in [43]. The outcomes reported a reduction of the grinding force and reduction of the tool damage in the assisted processes. Likewise, Trotta et al. [44] realized the innovative modification of microinjection molding through the proposal of detachable and personalized inserts. Such inserts were engineered using high-resolution femtosecond laser and µEDM machining processes. Laser micromachining was capable of processing, with increased removal rates, large surface areas with comparatively small depth attribute, while µEDM, was employed to attain high aspect ratio structures owing to the low removal efficiency.

**Figure 1:**
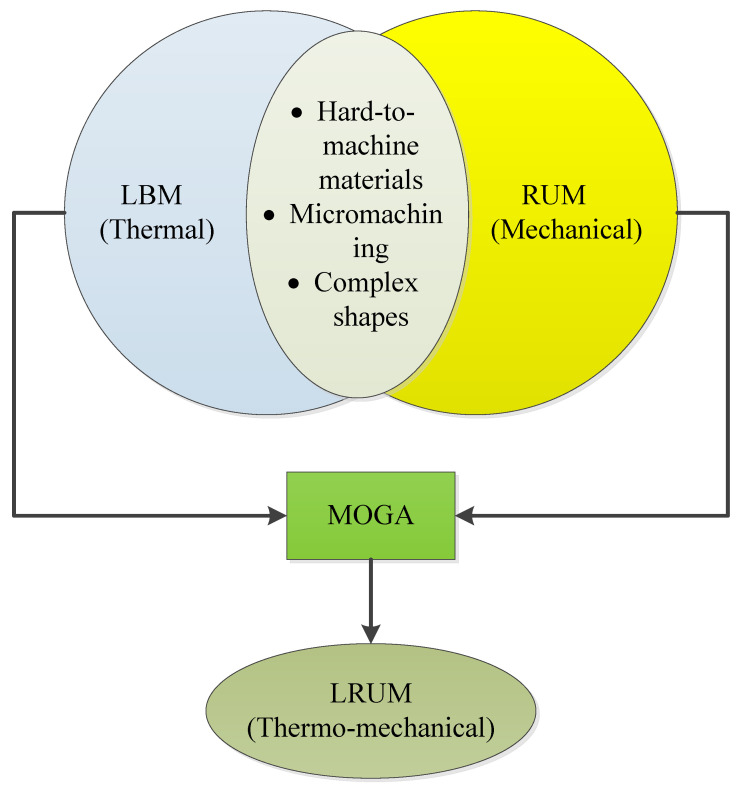
Unification of laser beam machining (LBM), rotary ultrasonic machining (RUM), and laser rotary ultrasonic machining (LRUM) processes.

The literature survey conspicuously demonstrates that the utilization of bioceramics in industries is hampered by the intricacy, high costs, and prolonged time involved with their machining. Thus, more cost-effective methods of machining bioceramics should be implemented. LBM and RUM are effective and reliable means for precision micromachining of bioceramic materials. Nonetheless, they both have their respective fundamental issues. LBM has problems with thermal damage, high surface roughness, and vulnerable dimensional precision [45,46,47]. RUM is also associated with high machining costs and low MRR [48]. To surpass their limits, a combination of LBM and RUM processes, known as laser ultrasonic rotary machining (LRUM), has been employed in the current study, as shown in Figure 1. The novelty of this work is that it addresses the challenge of machining microchannels (≤800 × 800 μm) in hard-to-machine bioceramics. Until now, according to the authors, very few studies have been published on the micromachining of biolox forte bioceramics. This work introduces an additional understanding of using LRUM to process these challenging-to-machine materials for even further reduction in machining costs and surface roughness. Furthermore, the impetus underlying the combination of the LBM and RUM processes stems from the fact that these two methods have many parallels including (i) both processes can be utilized to machine materials that are hard to cut like glass, ceramics, etc., (ii) both LBM and RUM can be employed to generate microfeatures, and (iii) can be adapted to machine complex shapes.

The following steps are adopted while executing the methodology of LRUM in this investigation: the LBM under its optimized parameters is first used as a prevailing machining method to produce microchannels in alumina bioceramic. It has been utilized to produce the substrate’s bulk geometry owing to its high MRR and lower machining costs. In the subsequent step, the RUM under its streamlined process conditions, is used as a finishing tool to finalize the geometry of premachined microchannels. The reasons for employing RUM to formalize the geometry are high dimensional precision and low tool wear. Furthermore, in this analysis, the different sizes of the microchannels are examined. Multi-objective genetic algorithm (MOGA) is used to optimize fundamental input parameters for both LBM and RUM processes. The performance indicators, namely dimensional accuracy of the channels including depth error (DE) and width error (WE) as well as surface quality including surface roughness and surface morphology are explored. Additionally, the correlation between the tool wear of RUM and LRUM methods is also analyzed.

## 2. Material and Method

Biolox forte ceramic from CeramTec, Plokhin root, Germany was chosen as the workpiece material. It consists of ultra-pure aluminum oxide ceramic (Al_2_O_3_, 99.97) and meets the highest standards of biocompatibility, longevity, and dimensional stability [49]. The dimensions of all the biolox forte samples used in the experiments were 50 mm in length, about 10 mm wide, and 10 mm thick. Table 1 details the mechanical and thermal properties of biolox forte as per the supplier [50].

**Figure 2:**
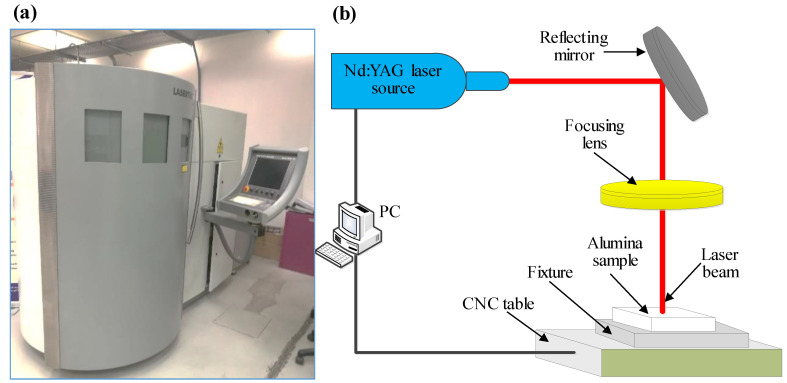
(**a**) Lasertec 40 machine, (**b**) schematic diagram of the laser system machine.

In this study, Lasertec 40 from Deckel-Maho-Gildemeister (DMG) Mori, Billefield, Germany was considered as a primary process for the manufacture of the microchannels in biolox forte ceramic. Lasertec 40 (see Figure 2a) is commonly used with its preset conditions, including 30 μm laser beam spot size, 30 W maximum power, 1064 nm continuous wavelength, and Nd: YAG pulsed mode. The laser with a spot size of 30 μm follows the Gaussian mode and emits a laser intensity of 42,441 kW/mm^2^ as a result of a pulse train with a pulse length of 10 μm and pulse duration of 10 μs. The illustrative working principle of Lasertec 40 is depicted in Figure 2b.

**Figure 3:**
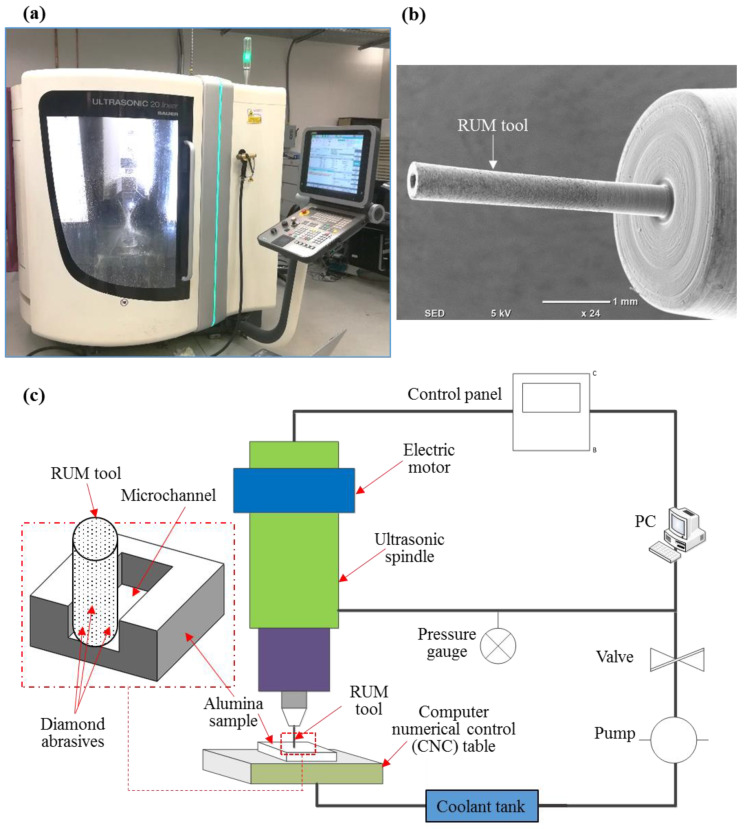
(**a**) Ultrasonic 20 Linear machine, (**b**) MicroRUM tool; (**c**) Schematic diagram of RUM.

Ultrasonic 20 linear which is used to finish the premachined microchannels also comes from DMG Mori, Billefield Germany (see Figure 3a). It is a precision machine with five axes that is predominantly used for precise micromachining and finishing operations. In the current investigation, microRUM tools with an outer diameter of 0.8 mm and 0.5 mm given by Schott Company, Mainz, Germany are used. Figure 3b displays the microRUM tool sample and Figure 3c portrays RUM schematics along with the 3D schematics of the produced microchannels.

**Figure 4:**
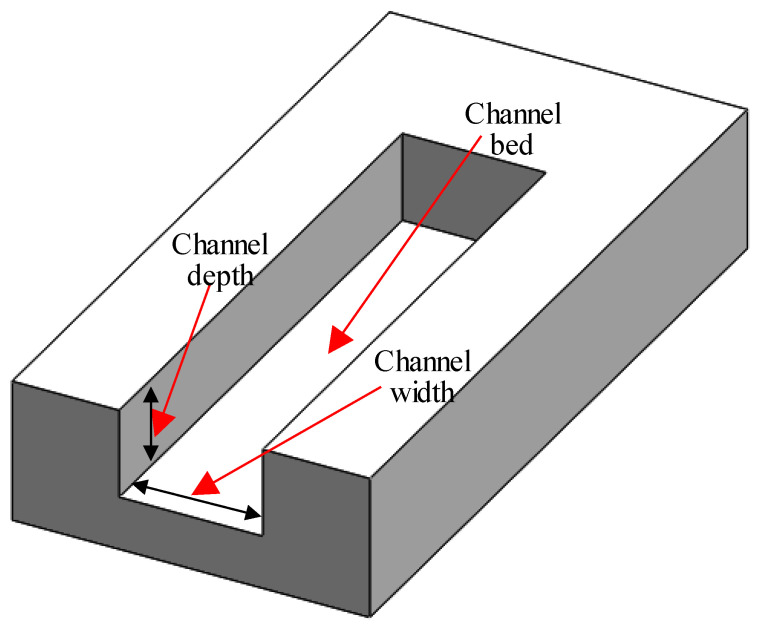
Schematic of microchannel.

Microchannels were produced in five varying sizes and two kinds of the cross-sectional area comprising square and rectangular cross-sections. Table 2 enumerates the dimensions of the desired channel sizes. The channel sizes were chosen depending on the requirements of microfluidics as stated in [51,52,53]. The microchannel schematic can be seen in Figure 4.

### 2.1. Machining Conditions

**Figure 5:**
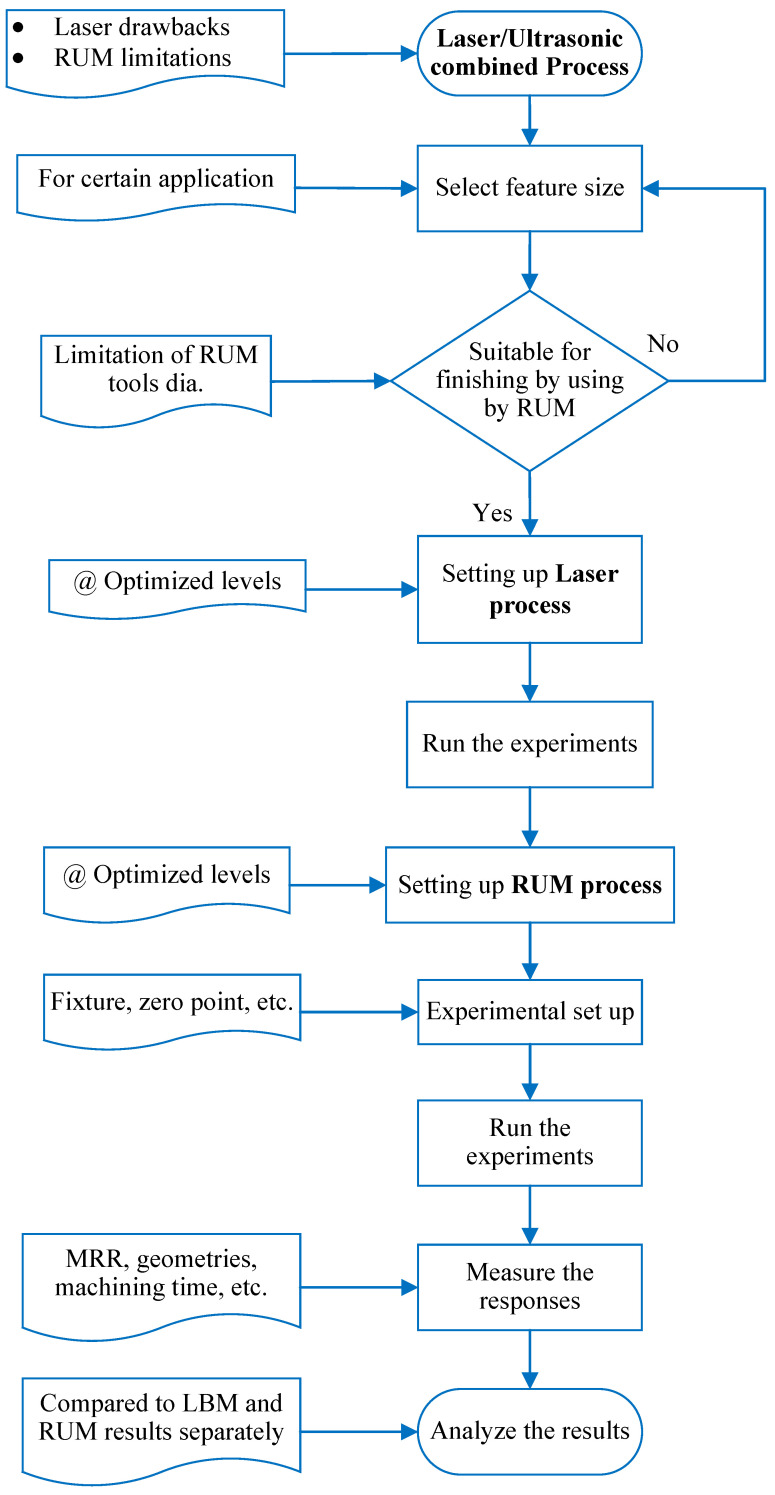
Workflow adopted in this study.

Before the final experiments were implemented, the selection of the laser and RUM key input parameters was essential. In this analysis, the process parameter ranges were specified based on previous studies of laser machining [45,47,54,55,56,57] and RUM [58,59,60,61,62]. The preliminary experiments were also conducted to establish the exact levels of the process parameters which were later used for the final experiments. Table 3 demonstrates the parameters of LBM, RUM input factors, and their levels used in the experiments. The entire workflow is described in Figure 5.

**Figure 6:**
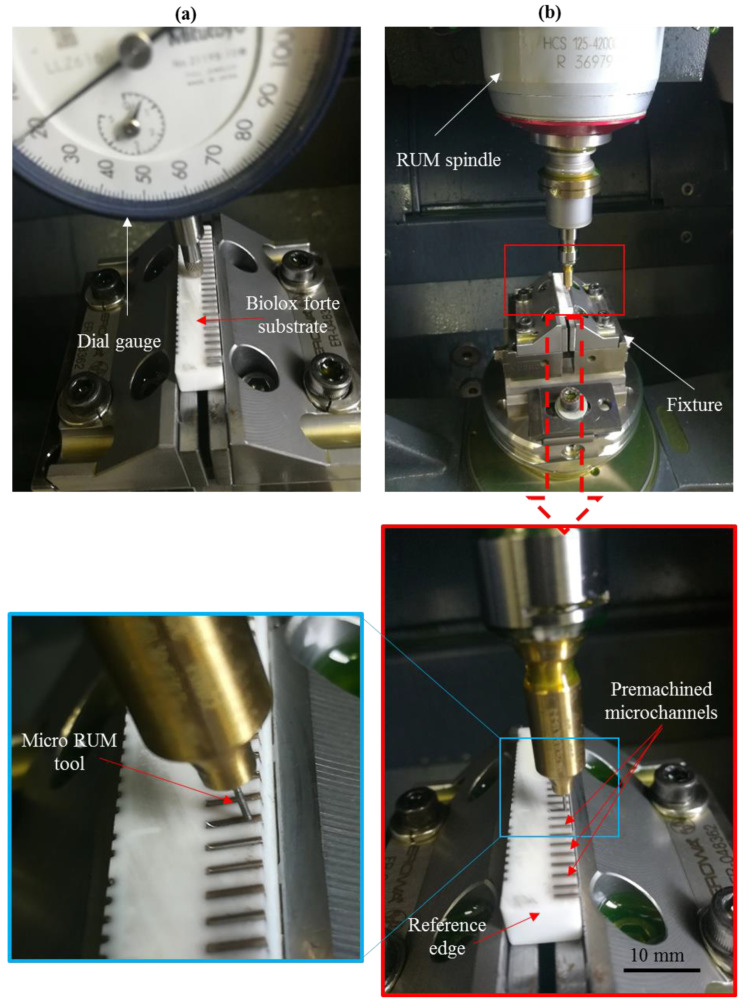
LRUM setup: (**a**) setup of workpiece leveling, (**b**) setup of tool alignment.

It should be emphasized that the method of machining using RUM in the premachined microfeatures was challenging due to the accompanying reasons. For example, the placing of the microRUM tool in the center of the premachined microchannel, the machine’s positioning accuracy, and the precision of the fixtures used. To resolve the above difficulties, the dial guage was used to establish the leveling of the workpiece, as shown in Figure 6a. In addition, one edge in Figure 6a of the workpiece that is the reference edge was chosen as the reference to direct the tool to the center of premachined microchannels as illustrated in Figure 6b. The workpiece measurements were determined automatically using a contact probe tool with a positioning error of ±2.5 μm. Additionally, the RUM microtools length and diameter were calculated and recalibrated using the integrated DMG laser tool length calibration method.

### 2.2. Multi-Objective Genetic Algorithm 

It should be noted that twenty-five microchannels of each size were machined by using LBM and RUM following the design of experiment tables. The MOGA was used to minimize the surface roughness (R_a_ and R_t_) and the dimensional errors (DE and WE) of the milled microchannels. The results of the optimization for each process were subsequently applied in the LRUM methodology. The chosen parameters of MOGA are depicted in Table 4.

### 2.3. Measurement Method

**Figure 7:**
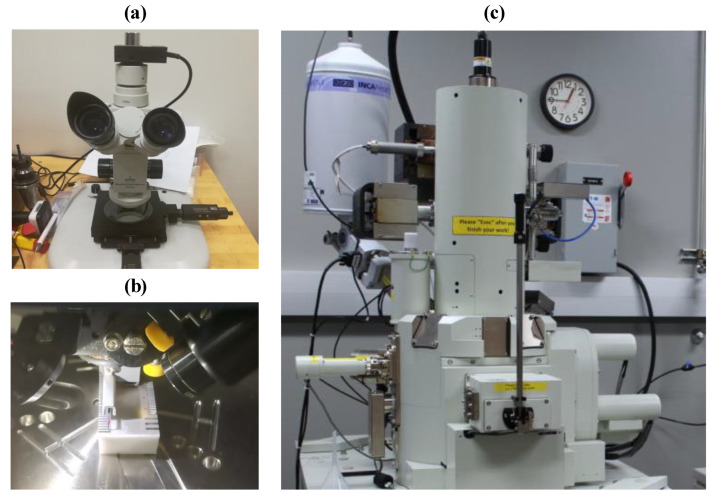
Measurement equipment: (**a**) optical microscope, (**b**) 3D profilometer, (**c**) SEM.

The geometries of the channels, including channel width and depth of channels (see Figure 4), were estimated using an optical microscope (see Figure 7a). Subsequently, by using Equations (1) and (2) respectively, the percentage of DE and WE was used for further analyses.

(1) $$\mathrm{WE}\%=\left|\frac{MW-DW}{DW}\right|\times 100$$

(2) $$\mathrm{DE}\%=\left|\frac{MD-DD}{DD}\right|\times 100$$

where *MW* is the estimated channel width following machining and *DW* is the required width, *MD* is the computed depth after machining, and *DD* is the desired depth. For example, 500 μm and 300 μm respectively, are the ideal width and depth for the channel size of 500 µm × 300 µm.

A 3D profilometer (DektakXT Stylus Profiler) from Bruker, MS, USA, was utilized to evaluate surface roughness by estimating arithmetical mean roughness (Ra) and a maximum height of the roughness profile (Rt) across every channel bed at six different spots. In this study, the Ra is utilized to quantify the surface roughness value because it has been the most common measure in the majority of the work that associates surface fatigue or failure to surface roughness. In medical applications also, that require microchannels, Ra is considered as the most important indicator of surface roughness [7,15]. The authors also measured some other roughness amplitudes such as Rq, Rv, and Rp. Hwoever, the obtained results followed the same trend as of Ra (Rq ≈ 1.3 Ra). Furthermore, the Ra is preferred over the root mean square (RMS) because Ra offers a holistic interpretation of the height variations of the surface and is less sensitive to high hills and troughs. In contrast, RMS is prone to the large peaks and valleys, where even a single high peak or imperfection within the microscopic surface morphology can elevate the RMS value more than the Ra value. For analysis, the mean of the five measurements is considered. Figure 7b presents the arrangement for determining the surface roughness. The microchannels were platinum-coated by including a thickness of 10 μm using JEOL Ltd.’s (Tokyo, Japan) JFC 1600 auto-fine coater to increase their clarity during electron microscopy scanning (SEM) study. A JEOL JEM-7500F scanning electron microscope (SEM), (JEOL, Tokyo, Japan) was used to investigate the surface morphology of microchannels as shown in Figure 7c. The machining time (MT) for all processes under analysis was determined by employing integrated MT measurement in both LBM and RUM.

## 3. Results and Analysis

**Figure 8:**
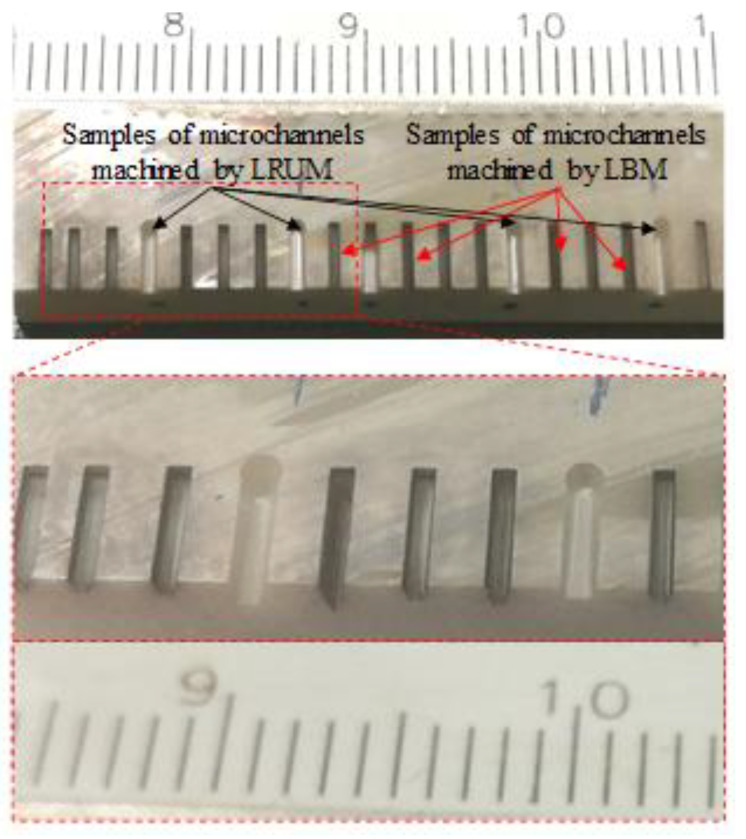
Samples of microchannels fabricated under the optimal conditions of LBM and LRUM.

The five distinct sizes of microchannels were produced in biolox forte ceramic materials under optimal laser ablation conditions. The fabricated channels were subsequently postprocessed through RUM in the combined process. Microchannel samples produced under optimum LBM and LRUM conditions can be realized in Figure 8. The following paragraphs address the combined process results compared to the independent LBM and RUM processes.

### 3.1. Surface Roughness

**Figure 9:**
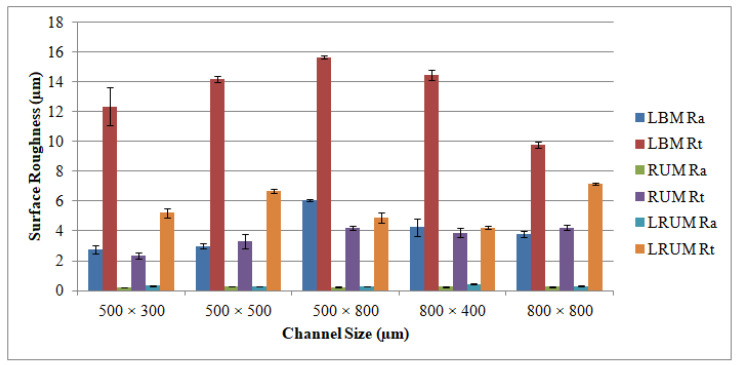
Results of surface roughness of the fabricated channels by using LBM, RUM, and combined LRUM processes.

**Figure 10:**
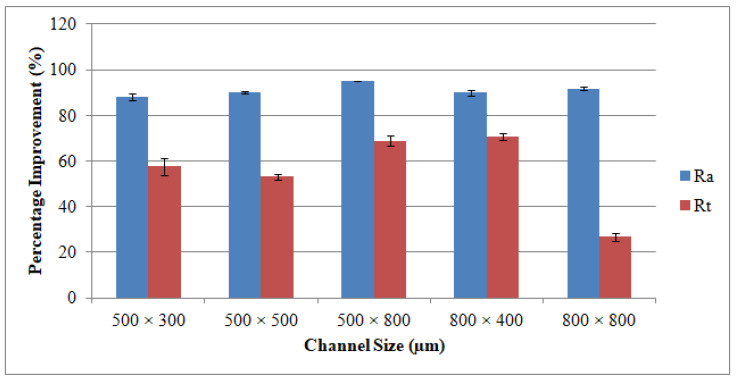
Percentage of improvement in the surface roughness of the fabricated channels by using LRUM comparing to LBM.

The plots shown in Figure 9 outline the surface roughness (Ra and Rt) of the different channels. The reported surface roughness estimates reflect the average measurements recorded at six random locations throughout the LBM scan directions and across the feed direction of the RUM tool. It could well be said that the Ra values extend from 2.76 to 6.06 μm and the Rt values vary from 9.82 μm to 15.67 μm for all channels produced in a biolox forte material using the LBM process. These values of roughness (Ra and Rt) can be attained at a pulse frequency of 5–7 kHz, laser intensity of 93–96 percent, and 100–200 mm/s of laser scanning speeds. The explanation for this finding is that the improvement in the pulse frequency results in a reduction of the laser energy while other parameters are unchanged. The lower laser power allows more unmolten materials to collect on the bed and sides of the ablated channel making the surface of the channel rougher. The roughness of the surface has been discovered to drop as laser intensity increases. It implies that low levels of intensity for ceramic material correlate to a nonuniform withdrawal of material which results in poor surface quality. It has also been noticed that the average temperature of the ablated channels decreases at greater levels of laser scanning speed (300 mm/s–400 mm/s). As a result, the molten content reduces and therefore the channel surface becomes rougher. Concerning the relationship between the channel size and the Ra (and Rt) values, the surface roughness becomes rougher when the channel depth increases in unchanged width. For instance, Ra values rise from 2.76 μm to 6.06 as the channel depth increases from 300 μm to 800 μm at a fixed width of 500 μm. This is because once the machined channel depth deepens, the laser loses its energy and it is harder to efficiently extract the fused mass, thereby creating the rougher surface. However on the other side, when the width increases to 800 μm the surface of the channel is smoother (Ra = 3.78 μm). Concerning the surface roughness of the RUM channels, the Ra and Rt values are found to be lower than those of the LBM outcomes. All the obtained values of Ra were less than 0.3 µm for all the fabricated channels by using RUM. There is no interesting correlation or connection between channel size and surface roughness for the channels formed by RUM and combined processes. A higher spindle speed of 7000 rpm, a lower feed rate of 0.4 mm/min, a cutting depth of 0.025 mm, a medium to high vibration amplitude of 20–25 µm and a 20–25 kHz frequency were used to achieve the Ra and Rt values. In general, it can be inferred from Figure 9 that the LRUM findings are close to the RUM responses. While comparing LBM with LRUM performance, the surface roughness has improved significantly by more than 88 percent for Ra and 26 to 72 percent for Rt in all channel sizes in the case of the LRUM combined process. For example, as shown in Figure 10, for the 800 µm × 400 μm size channel the Ra is decreased by 90 percent and the Rt by 72 percent. The uncertainties responsible for variation in the surface quality of different channels (acquired using LRUM) has been quantified using standard deviations (SD). The SD is represented using error bars in Figure 9 and Figure 10. There can be many uncertainties, such as tool wear, measurement errors, vibrations, operator experience, etc., that caused variance in the surface quality.

**Figure 11:**
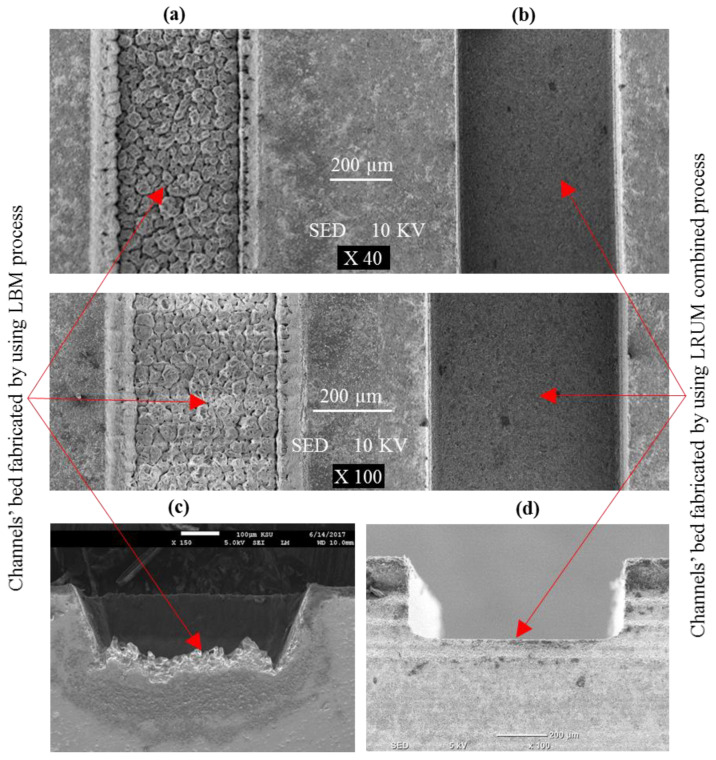
Comparison of the surface morphology of the fabricated channels by using LRUM comparing to LBM, (**a**,**b**) channels’ bed, (**c**,**d**) channels’ side.

The analogy of the surface characteristics of a channel generated by LBM and LRUM processes is illustrated in Figure 11. Figure 11a,b shows the bed of the channel while Figure 11c,d represents the sides of the channel. The surface produced through the LRUM process is observed to be very smooth across the channel’s sharp edge. However, a rough surface morphology can be observed in the case of the channel generated by LBM. It is quite common for LBM parts to have a higher surface roughness. For example, in [63] the roughness (Ra) of the microchannels machined by using the Nd:YAG laser process reached up to 15 µm for the smaller channel size and 4 µm for the bigger channel size (1000 µm width). In another study, it was found that the surface roughness (Ra) varied between 1.5 µm and 4.8 µm for the microchannels machined in zirconia ceramic by the laser ablation process [55]. The surface roughness in terms of Ra was also found to be between 4.5 µm and 12 µm for the microchannels machined in alumina [64]. Generally, the higher surface roughness with LBM can be attributed to its high laser heat impact in proportion to the thickness of the test specimen. Additionally, the higher thermal damage and formation of the recast layer are the other reasons for poor surface roughness in LBM [65]. The rapid cutting speed (to achieve higher MRR) for LBM has indeed been the primary reason for its use in this research. This high speed, however, comes at the price of adverse and unfavorable surface integrity outcomes. The similar behavior of LBM was also reported by Holmberg et al. [66], who evaluated the performances of EDM, LBM, and abrasive water-jet machining. They found the highest surface roughness was for LBM amongst the three approaches. According to them, LBM could be an option for thin workpieces of less than 8 mm, but rigorous surface integrity investigations are then needed to assess the effect and the necessary postprocessing. It must also be acknowledged that the generated depth of the channels is more than the target depths in some channel sizes. The LRUM is unable to machine extra depth to maintain the dimensional accuracy of the channels as per the planned one, resulting in higher Ra and Rt values than the RUM results.

### 3.2. Dimensional Accuracy

**Figure 12:**
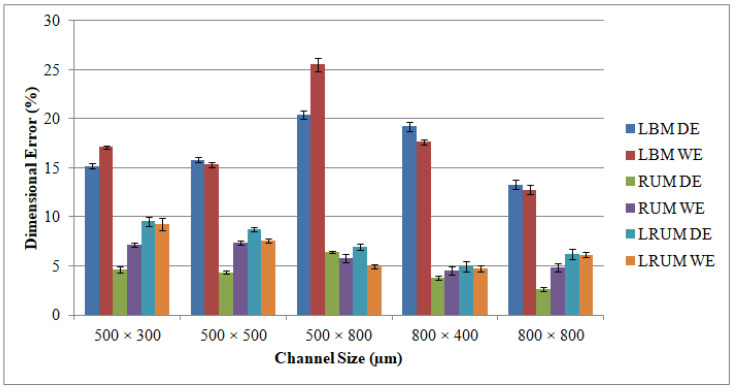
Results of dimensional errors of the fabricated channels by using LBM, RUM, and combined LRUM processes. DE and WE: dimensional errors.

The graphs appear in Figure 12 encompass details on dimensional accuracy for different channels in terms of DE and WE. It must be emphasized that the channel width presumed in this study is the bottom width (see Figure 4), because the top widths produced have been found to meet the intended widths for all channel sizes. It simply indicates that there is no issue with the shielding effect of the molten material on the top surface and that the laser provides completely focused energy. In addition, the problem of tool wear is not detected at the beginning of machining the first cutting depth in RUM. In LBM, the DE is discovered to be positive (overcut), i.e., if the channel depths are lower than the channel widths, the depths created are larger than the depths intended. It appeared at channel sizes of 500 μm × 300 μm, 500 μm × 500 μm, and 800 μm × 400 μm. In contrast, the DEs are spotted to be negative (undercut), i.e., the depths produced are smaller than the expected depths if the depths of the channel are higher than the channel widths such as the channel size of 500 μm × 800 μm. This is because the laser loses its energy when the machined depth becomes deeper. Specifically, with an increase in channel depth, the DE increased from 15.2% for a channel size of 500 μm width × 300 μm depth to reach approximately 20.5% for a channel size of 500 μm width and 800 μm depth.

**Figure 13:**
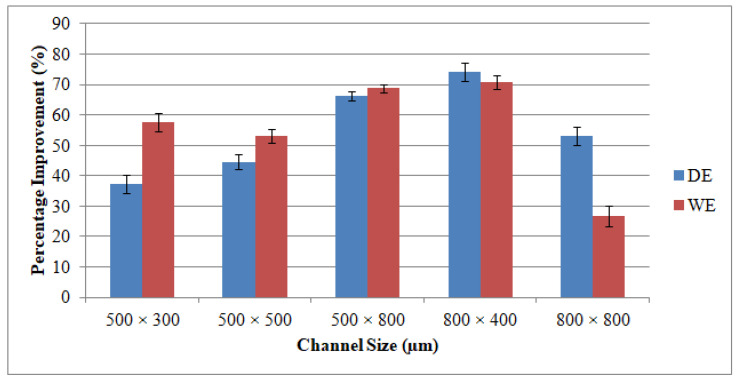
Percentage of improvement in the dimensional accuracy of the fabricated channels by using LRUM comparing to LBM.

Taking into consideration LBM’s WE, it is noticed that the width values acquired for all channel sizes are below the anticipated widths. This is due to the inherent properties of the laser that it invokes some tapering as when the machining depth proceeds. The WE is larger than the DE in most manufactured channels. The higher WE can be noticed in the deeper channel size (500 μm × 800 μm). However, the minimum WE equivalent to 12.8 percent could be figured at the channel size of 800 μm × 400 μm. The DE and WE of RUM are always positive. This is because the outer diameter of the RUM tool (0.5 mm and 0.8 mm) is the tool’s nominal diameter. The effective diameter of the tool is greater than the nominal diameter due to the variation in the abrasive sizes of the diamond bonded to the tool. Abdo et al. [62] also observed and explained this phenomenon of overcutting during RUM because of the discrepancy in the grit size of the diamonds. With respect to the channels produced using the LRUM process, it is discovered that the DE and the WE are less than 10 percent for all the machined microchannel sizes. The increase in the percentage DE and WE of LRUM as opposed to the performance of LBM for all channel sizes can be realized in Figure 13. For example, using the LRUM procedure it is found that the DE and WE values in the 800 μm × 400 μm size channel are reduced by 74 percent and 72 percent respectively. The uncertainties that caused variation in DE and WE of different channels are also quantified using SD. The SD of dimensional errors is depicted in Figure 12 and Figure 13 utilizing error bars. There can be many uncertainties, including tool wear, measurement errors, vibrations, fixtures, ambient temperature, etc., that have produced variance in the dimensional errors.

**Figure 14:**
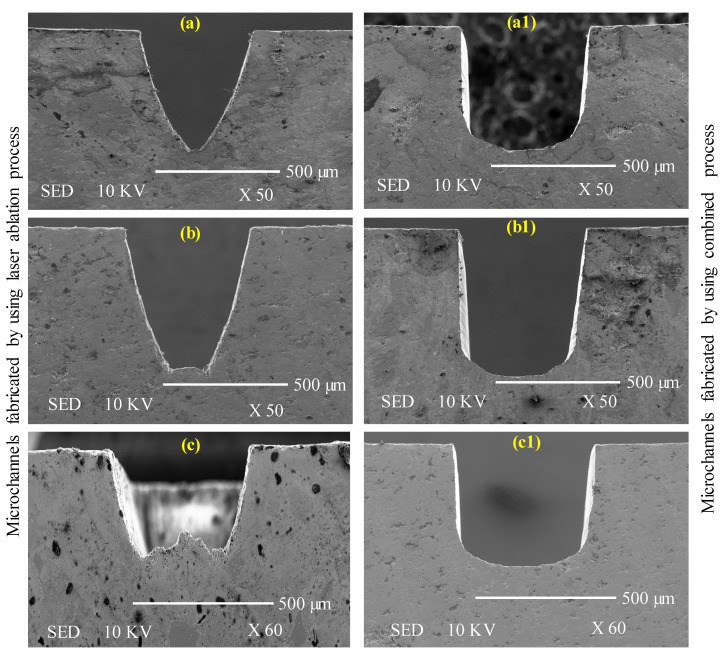
Comparison of the cross-sections of the fabricated channels by using LBM and combined LRUM processes: (**a**–**c**): cross-sections of the microchannels machined by using LBM process; (**a1**–**c1**): cross-sections of the microchannels machined by using LRUM combined process.

The illustration of cross-sections of channels obtained through the LBM and LRUM processes is provided in Figure 14. The cross-sections accomplished by the LRUM process correctly approximate the rectangular or square cross-section as perceived in the design phase. However, cross-sections manufactured by LBM have deviated significantly from their actual representation. As shown in Figure 14, the channel cross-section ended up taking the form of a conical or tapered cross-section rather than a rectangular or square cross-section. It implies the bottom width produced in LBM is smaller than expected. This can be due to the inherent characteristics of the laser that it imparts some taper as the machining depth progresses [45]. Certainly, the LBM could not machine or transfer the necessary energy to maintain the channel’s dimensional accuracy according to the expected one. LRUM has overcome this deficiency of LBM through RUM to precisely generate the required channel cross-sections.

### 3.3. Machining Time

**Figure 15:**
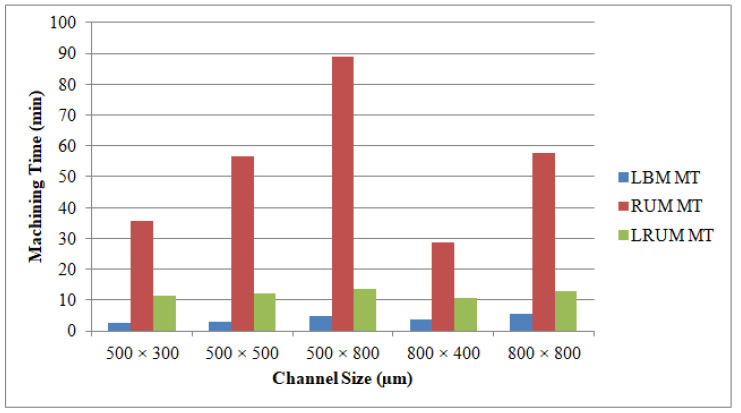
Results of the machining time (MT) of the fabricated channels by using LBM, RUM, and combined LRUM processes.

In most machining practices, MT is among the most important considerations, because it regulates overall machining costs. In the LBM method, laser intensity and laser scanning speed are the key drivers impacting the MT at a constant layer thickness. Furthermore, the feed rate and depth of cut are the most major factors influencing the MT in RUM. Figure 15 shows the outcomes of the mean MT for various channels produced using LBM, RUM, and LRUM methods.

**Figure 16:**
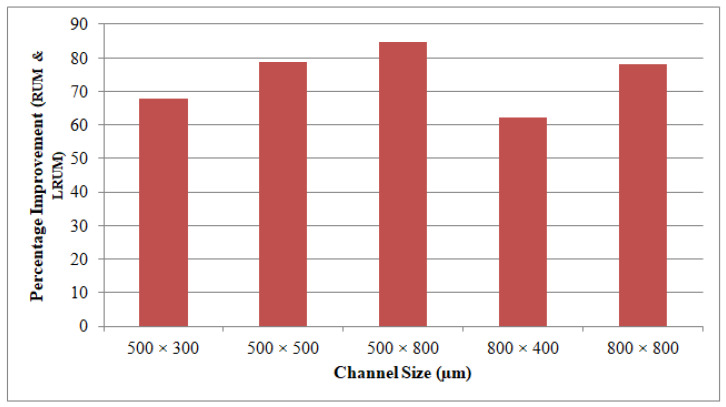
Percentage of improvement in the MT of the fabricated channels by using LRUM comparing to RUM.

The LBM process contributes to the least MT amongst the three machining technologies that were deployed. Even though the LRUM process produced a higher MT relative to the LBM process, it is substantially superior in terms of MT as opposed to RUM. For instance, in the LRUM procedure, the MT decreases from 84.9 min (in RUM) to 12.7 min. The selected channel widths are equal to the outer diameters of the RUM tool in the RUM process. The 800 μm tool diameter is more robust than the 500 μm tool and depending on RUM’s optimum process parameters the selected feed rate and cutting depth are greater than those of the small size tool (500 μm diameter). Therefore, the MT for the channel sizes of 800 µm × 400 µm (26.8 min) and 800 µm × 800 µm (53.4 min) are smaller than 500 µm × 300 µm (3.8 min) and 500 µm × 800 µm (84.9 min) respectively. The percentage of changes made in the MT by using the LRUM process as opposed to the RUM process’s MT can be observed in Figure 16.

### 3.4. Tool Wear

**Figure 17:**
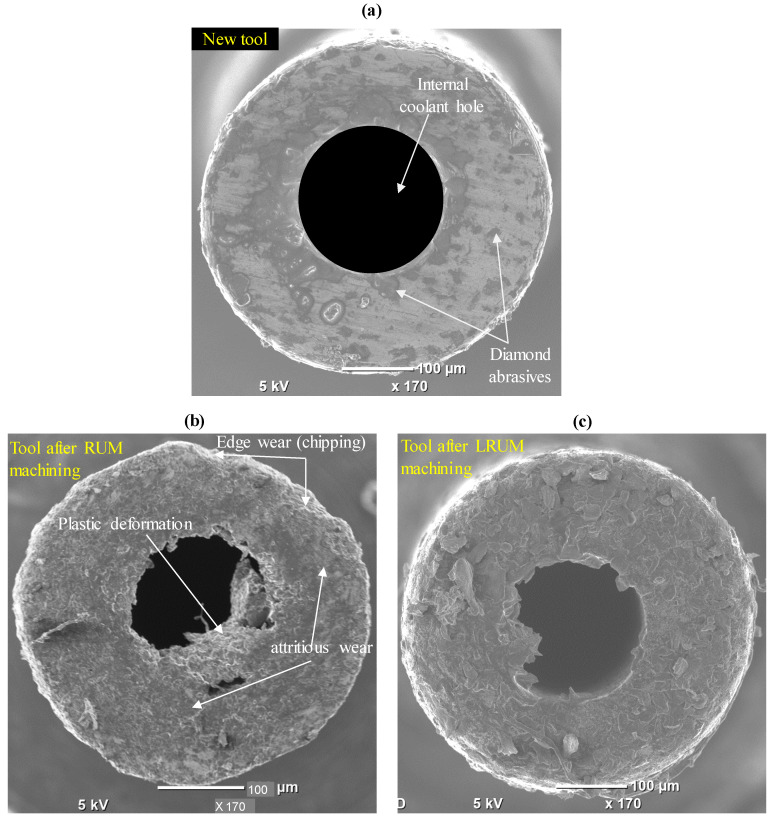
Comparison of the RUM tool (**a**) before machining (new tool), (**b**) after machining by using RUM, (**c**) after machining by using LRUM.

The comparisons of the new RUM tool, the tool after machining twelve channels by using RUM, and the tool used for finishing twelve channels by using LRUM processes are depicted in Figure 17. Figure 17a provides the end-cutting-face perspective of the new tool, in which the diamond abrasives and bonding material can be easily viewed. It implies that the tool exhibits uniform inner and outer radii as well as a fairly sharp cutting edge. The end cutting edge of the tool used in the LRUM process is shown in Figure 17b in which attritions wear, edge chipping, and rounding of tools can be observed, and these wear mechanisms have also been documented in [67,68]. In addition, it is clear in Figure 17b that, due to the increased pressure on the tool end face during machining, some of the tool bonding material along with the diamond abrasive are plastically deformed and dragged through the coolant hole. Such tool wear can adversely affect the coolant efficiency of the tool owing to the coolant passage obstruction. Figure 17c shows the end cutting edge of the tool used for finishing twelve channels by using the LRUM process. It can be noted that there is a minimal rounded edge and less plastic deformation during machining under the LRUM process. It must be mentioned that the length of the tool is recalibrated by the integrated DMG laser tool length calibration device after every microchannel is machined.

## 4. Conclusions

Microchannels of varying sizes were manufactured in biolox forte ceramic material using LBM, RUM, and LRUM processes at their optimal parameters. The MT is far lower during LBM but the performance of the microchannels is not reasonable in terms of surface finish, surface morphology, and dimensional precision. However on the other side, the surface quality and dimensional accuracy of the RUM-produced microchannels are indeed very high, but it is a slower process because the MT is very high. To solve these issues and maximize the benefits of both the machining processes (less MT of LBM in addition to higher surface quality and dimensional accuracy of RUM), a new combined machining process is introduced, known as the LRUM process. The microchannels are initially machined using LBM in LRUM, where much of the material is extracted and after which the exact microchannels are completed with RUM. While using LRUM the surface quality has been improved in terms of both surface roughness and surface morphology for microchannels of different sizes compared to LBM findings. The Ra values are reduced by more than 88 percent for all sizes of generated microchannels. It is also reported that in some channel sizes the DE and WE values are also decreased by 70% and 80% respectively. Moreover, the MT of RUM is reduced by 85% with the execution of LRUM, particularly in the channels with greater depth (size of 500 µm width × 800 µm depth). The tool wear is also found to be reduced while using combined LRUM as compared to the tool wear resulting from RUM under the same machining conditions. Certainly, the overall machining cost is reduced considerably with the application of LRUM.

## Figures and Tables

**Table 1:** Mechanical and thermal properties of biolox forte.

Property	Value (Unit)
Fracture toughness	3.3 (MPa·m^1/2^)
Compressive strength	5500 (MPa)
Tensile strength	665 (MPa)
Bulk density	3.98 (g/cm^3^)
Poisson’s ratio	0.22–0.25
Vickers hardness	1900 (HV 1)
Young’s modulus	413 (GPa)
Thermal conductivity 20 °C	30 (W/mK)
Thermal expansion coefficient	5.4 (10^−6^ 1/K)
Melting point	2277 (°F)

**Table 2:** Dimensional specification of microchannels used in this study.

Cross-Sections	Channel Size (µm)	
	Depth	Width
Rectangular	500	300
	500	800
	800	400
Square	500	500
	800	800

**Table 3:** LBM and RUM parameters and their respective levels.

LBM Input Parameters	Range	RUM Input Parameters	Levels
Laser intensity	88%–96%	Spindle speed	2000–7000 rpm
Scanning speed	100–400 mm/s	Feed rate	0.4–1 mm/min
Pulse frequency	5–12 kHz	Depth of cut	0.025–0.1 mm
Layer thickness	2 µm	Vibration amplitude	5–25 µm
Track displacement	10 µm	Vibration frequency	20–32 kHz

**Table 4:** MOGA parameters used in this study.

Parameter	Value
Number of generations	100
Probability of direction cross-over	0.5
Probability of selection	0.05
Probability of mutation	0.1
DNA string mutation ratio	0.05
Random generator seed	1
